# Model-Derived Dispersal Pathways from Multiple Source Populations Explain Variability of Invertebrate Larval Supply

**DOI:** 10.1371/journal.pone.0035794

**Published:** 2012-04-25

**Authors:** Carla P. Domingues, Rita Nolasco, Jesus Dubert, Henrique Queiroga

**Affiliations:** 1 Centro de Estudos do Ambiente e do Mar (CESAM) and Departamento de Biologia, Universidade de Aveiro, Campus Universitário de Santiago, Aveiro, Portugal; 2 Centro de Estudos do Ambiente e do Mar (CESAM) and Departamento de Física, Universidade de Aveiro, Campus Universitário de Santiago, Aveiro, Portugal; University of Canterbury, New Zealand

## Abstract

**Background:**

Predicting the spatial and temporal patterns of marine larval dispersal and supply is a challenging task due to the small size of the larvae and the variability of oceanographic processes. Addressing this problem requires the use of novel approaches capable of capturing the inherent variability in the mechanisms involved.

**Methodology/Principal Findings:**

In this study we test whether dispersal and connectivity patterns generated from a bio-physical model of larval dispersal of the crab *Carcinus maenas*, along the west coast of the Iberian Peninsula, can predict the highly variable daily pattern of wind-driven larval supply to an estuary observed during the peak reproductive season (March–June) in 2006 and 2007. Cross-correlations between observed and predicted supply were significant (p<0.05) and strong, ranging from 0.34 to 0.81 at time lags of −6 to +5 d. Importantly, the model correctly predicted observed cross-shelf distributions (Pearson r = 0.82, p<0.001, and r = 0.79, p<0.01, in 2006 and 2007) and indicated that all supply events were comprised of larvae that had been retained within the inner shelf; larvae transported to the outer shelf and beyond never recruited. Estimated average dispersal distances ranged from 57 to 198 km and were only marginally affected by mortality.

**Conclusions/Significance:**

The high degree of predicted demographic connectivity over relatively large geographic scales is consistent with the lack of genetic structuring in *C. maenas* along the Iberian Peninsula. These findings indicate that the dynamic nature of larval dispersal can be captured by mechanistic biophysical models, which can be used to provide meaningful predictions of the patterns and causes of fine-scale variability in larval supply to marine populations.

## Introduction

Most marine populations are demographically open, as individuals are regularly exchanged among sets of local populations, either through the transport of planktonic propagules by currents or by the movement of adults [1], [2]. Consequently, local populations of marine species depend, in part, on processes that occur elsewhere that influence the production, movement, and survival of dispersers. Understanding how networks of populations (i.e., metapopulations) are connected by dispersal is therefore crucial for understanding gene flow, evolution, biodiversity patterns and resilience of marine populations [3] as well as for developing effective management measures capable of providing local benefits.

In the case of benthic marine animals, where the adult is sessile or sedentary, dispersal is accomplished by a planktonic larva. Although results from theoretical modelling [1], genetic studies [4], [5], [6], [7], [8], and the use of site-specific natural tags [9], [10] suggest that moderate levels of larval exchange over a range of spatial scales likely typifies many marine metapopulations, the largely unbounded aquatic medium where larval dispersal takes place and the small size of the larvae create insurmountable logistical challenges in obtaining direct empirical evidence to confirm these findings for most marine species. A further difficulty in obtaining empirical evidence of dispersal and connectivity is caused by eddy diffusion and by larval mortality. Turbulent mixing spreads larvae over larger areas, effectively redistributing the larvae over large volumes of the ocean [11]. Instantaneous mortality rates for marine larval stages are on the order of 0.0161 to 1.0100 [12]. Combined, these processes further decrease the likelihood of detecting larvae by conventional sampling methods, particularly ones that are dispersed large distances away from their population of origin [13].

In the present study we provide a novel test of the connectivity framework by: (1) generating 4-month empirical time series of daily larval supply of the shore crab *Carcinus maenas* to the Ria de Aveiro, an estuary on the west coast of the Iberian Peninsula during the peak reproductive seasons in 2006 and 2007, and (2) comparing these time series to predicted larval supply time series derived from a coupled oceanographic and biological numerical model (biophysical model). Because biophysical models provide a simulation environment integrating abiotic variability of the marine environment with biological processes at multiple temporal and spatial scales [14] they are a powerful tool to understand dispersal, supply and connectivity of populations in the ocean [15]. Biophysical models of larval dispersal have been used to provide theoretical expectations of the effects of various forcing factors on transport [16], [17], [18], [19] or to predict the resulting spatial distributions of adults [20], [21] recruits [22], or larvae, against *in situ* observations of larval concentration [17], [23], [24], [25]. Recently, biophysical models have also been used as basis for seascape genetic models and applied to explain phylogeographic patterns and spatial distribution of allele frequencies [26], [27]. A limitation of the biophysical larval dispersal models used so far is the appropriate choice of the response variable and of the spatial and temporal scales of the comparisons between the observations and the predictions of the model [15]. For instance, the use of settlement or recruitment data may not be appropriate because several physical [28] and biological [29] processes may decouple larval abundance in the water column from subsequent spatial densities of recruits. Additionally, a mechanistic understanding of the dispersal and supply processes requires data collected at daily time scales, which is the typical temporal scale of changes in larval abundance near settlement habitats [30], [31], [32].

In the present study, by recording larval supply at daily frequencies over the reproductive season, we were able to connect the temporal dynamics of the larval supply process with the spatio-temporal dynamics of the dispersal and connectivity process. To our knowledge, this is the first investigation into the linkages between larval dispersal and larval supply in a marine system. The simulations were performed using the Regional Ocean Modeling System (ROMS) and a Lagrangian particle-tracking submodel that includes advection and diffusion, as well as diel vertical migration, temperature-dependent growth and mortality. The observed megalopal supply series obtained during two years were compared with predictions of the model using a stepwise approach where we tested different behavioural, growth and mortality scenarios that progressively constrained potential dispersal distance. The average and maximum potential dispersal distances during the planktonic larval phase were then estimated. The model successfully predicted wind-driven supply with time lags of −6 to +5 d, with estimates of average dispersal distances ranging from 57 to 175 km. The strength of the predictions provides compelling evidence for the utility of biophysical models in general to produce dispersal pathways of individual larvae, allowing for testing of complex hypotheses about dispersal scales, demographic connectivity and gene flow, as well as providing fundamental ecological information necessary for management of natural resources and ecosystems in a cost-effective way.

## Methods

### Ethics Statement

Samples of larvae were collected with passive plankton nets deployed from a floating pier of the Clube de Vela da Costa Nova, located in the Canal de Mira, Ria de Aveiro. This area is included in the bird Special Protection Area (SPA) of the Ria de Aveiro, which is the only nature conservation legal diploma applicable to the area. Since collection of biological material with the use of plankton nets is not forbidden under the statutes of this SPA, permits are not required from the legal management body. Permits were however obtained from the direction of the Clube de Vela da Costa Nova to use the floating pier. *Carcinus maenas* is not an endangered or protected species. To the best of our knowledge, none of the unidentified zooplanktonic species that may be present in the samples is in any way endangered or protected.

### The biological model

We developed our biological model to mimic the early life-history characteristics of the portunid shore crab, *Carcinus maenas*, a keystone predator and invasive species that forms large populations in coastal ecosystems worldwide. In southern Europe *C. maenas* lives mostly in estuarine systems. The larval phase comprises four planktotrophic zoeae and one megalopa [33] that develop in the water column from four to six weeks, depending on water temperature [34]. Berried *C. maenas* females move to the lower estuary where larvae are released during nocturnal neap ebb tides following quarter moons, resulting in pulses of abundance of first zoeae that last for 3 to 5 days and recur at fortnight intervals [35], [36] from winter to early summer. First zoeae migrate to surface waters immediately after hatching and are exported from the estuary by the strong ebb currents, usually before the turn of the tide [36]. The megalopa is the stage that reinvades estuaries by selective tidal stream transport [37]. *C. maenas* larvae display diel vertical migration behaviour in coastal waters [36], [38] and their dispersal pathways deviate strongly from expectations based on assumptions made by passive dispersal models [39].

### Time series of supply

**Figure 1:**
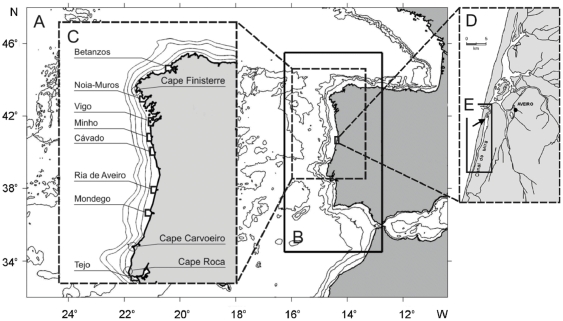
Map of the study zone and model domains. (A) first domain (FD); (B) large domain (LD); (C) location of estuaries used in the model showing the shelf area adjacent to each estuary where larvae were emitted and recruited; (D) Ria de Aveiro; and (E) Canal de Mira. (C) also shows details of the 50, 100, 250 and 500 m isobaths of the smoothed bathymetry used in the model. Arrow in (E) indicates location where the passive nets were deployed.

Time series of supply of *Carcinus maenas* megalopae were obtained in the Ria de Aveiro, a bar-built estuary located on the northwest coast of Portugal (Figure 1). Circulation in the Ria de Aveiro is dominated by tides, which are semidiurnal with an average range of 2.1 m. Collections of larvae were made with two passive plankton nets that were deployed daily from a floating pier in the Canal de Mira, Ria de Aveiro (Figure 1). These nets were designed to be continuously submersed, but to sample megalopae only during the flood tide, with a baffle inside the nets to prevent the loss of material during ebb tide. Both nets were deployed facing the inlet, one net below the water's surface and the other above the bottom, and both were recovered each morning for enumeration of megalopae. The sampling periods covered much of the species' larval season of two different years: March 7 to June 29, 2006, and March 11 to June 30, 2007. The data reported in the present paper are daily averages of the number of megalopae collected by the two nets. Field sampling methods and original data are fully described in [32], [40].

### The oceanographic model

The simulations were conducted using a 3-D free-surface, terrain-following primitive equation hydrostatic model configurable for fully realistic regional applications, based on the Regional Ocean Modeling System (ROMS) [41]. The present configuration represents an improvement and extension of the configuration used by Peliz *et al.* [42] and Oliveira *et al.* [43] to the Atlantic margin of the Iberian Peninsula. For a realistic simulation of the Western Iberian margin it is necessary to include local aspects like the Gibraltar Strait exchange (Mediterranean inflow/outflow) and the wind-driven dynamics. The remote circulation influencing the western limit of the study region associated with the Azores Current (AC) and the Iberian Poleward Flow (IPC) should also be accounted for. The large scale AC is related to the origin of water mass distribution observed along the western Iberian margin, and should be taken into account in order to obtain a good representation of the properties of the water masses in the region. The role of IPC is double: on one hand it is a mechanism of transport in the poleward direction, and on the other hand it represents a mechanism of retention for larvae [44], forming a kind of barrier to avoid offshore dispersal of larvae. To resolve the large and small scale circulation, two grids were used (Figure 1): a first domain grid (FD, Figure 1A), with a resolution of 1/10° (*ca.* 10 Km), from 32°W to 0.5°W and 30°N to 48°N. This FD was used to provide initial and boundary conditions, through offline nesting, to the large domain (LD, Figure 1B), which has a horizontal resolution of 1/27° (i.e. a mean resolution of 3.5 km), and 60 vertical levels, including the western Iberian margin, from the Gulf of Cadiz (34.5°N) to the Bay of Biscay 45.5°N, and from 12.5°W to the Strait of Gibraltar at 5.5°W. The LD covers an area of 1200×600 km and constitutes the target domain used for the dispersal simulations. The FD was first initialized from rest using monthly temperature and salinity climatologies from Levitus & Boyer [45] and Levitus *et al.* [46] at the boundaries, and was forced using monthly surface fluxes from Comprehensive Ocean-Atmosphere Data Set (COADS) [47]. Monthly geostrophic and Ekman velocities were applied along the open lateral boundaries. The methodology used is similar to that used in climatological studies of the coastal transition zone of the California current system [48]. This FD configuration reached equilibrium solutions after four years. At this stage, the Mediterranean Water (MW) was represented using a nudging term. After that period, and once the ocean reaches equilibrium, realistic forcing at the surface (instead of a climatological one) was used. The forcing consisted of the NCEP2 air-sea fluxes (www.ncep.noaa.gov) and QuikScat reanalyzed satellite winds from CERSAT (cersat.ifremer.fr) for the period 2001 to 2009, with a spatial resolution of 0.5°. The outputs of the FD were used to initialize and provide boundary conditions to the target domain, LD, through offline nesting. Forcing of the LD was the same as for the FD, ensuring consistency of forcing for both domains and avoiding problems at the boundaries. For the target domain, LD, the Mediterranean Undercurrent (MU), which originates at the Strait of Gibraltar and flows along the southwest and west margins of the Iberian Peninsula, was imposed by a boundary inflow/outflow condition at the Strait of Gibraltar [42]. The inflow of freshwater to the ocean originated from the main rivers of the region was included in the form of realistic river outflow (provided by INAG, Water Institute of Portugal), when available. When there were no registers of river outflow during a period of time a climatological value for seasonal river outflow was imposed. The outputs of the model, consisting of temperature, salinity, and three-dimensional velocity fields, were stored every four hours in order to be used for the Lagrangian module described below.

### The Lagrangian offline model

In order to simulate hatching, behaviour, growth and mortality of *Carcinus maenas* larvae an Individual Based Model (IBM) was coupled to ROMS using ROFF (ROMS Offline) [49], which is a drifter-tracking code that simulates larval trajectories from stored ROMS velocity and hydrological fields. The drifter-tracking code simulates larval trajectories from stored ROMS velocity and hydrological fields using a high order predictor corrector scheme to integrate the motion equation dX/dt = U_roms_(X,t), with X being the position vector (x,y,z), and U_roms_ being the modelled 3D velocity vector over time, given an initial condition X(t_0_) = X_0_. We first tested, on a subset of the simulations, that this offline procedure yields qualitatively and quantitatively similar results to the online procedure, in which case the larvae were advected with the time step of the model (300 s). Additionally to the advection generated by the model velocities, and similarly to Peliz *et al.* [24], the particle movements included random velocities in the vertical direction, which were used to parameterize unresolved turbulent processes. A diel vertical migration (DVM) scheme inferred from Queiroga [50] and dos Santos *et al.* [38] was also explicitly introduced in most of the model experiments. The DVM scheme consisted of forcing the larvae to drift at depth between 06 h and 20 h, and at the surface between 22 h and 04 h, every day, while during the remaining periods the larvae migrated between the surface and the deep levels (defined as the bottom layer of the model if the local depth is shallower than 60 m, or 60 m if the local depth is deeper).

Planktonic larval duration (PLD) and mortality rate caused by physiological stress from varying temperature and salinity were modelled pooling information from laboratory studies where larvae were reared without substrate [34]. The proportional effects of temperature on PLD, and of temperature and salinity on mortality, based on the time a larva was exposed to a specific temperature in the case of PLD, or to a specific combination of temperature and salinity in the case of mortality, were estimated by linearly interpolating between the laboratory data for each larval stage. Age and the probability of death were assessed at each time step of the model (300 s). Larvae were killed randomly based on the proportional death rate during the previous time interval. If a larva survived physiological stress it would grow from age 0 at hatching to 4 at the moult to megalopa, corresponding to stages zoeae 1 to 4; megalopae lived and remained competent until age 5 and then died. No other temporally or spatially distributed source of mortality (e.g. predation) was used because of lack of information.

In order to simulate hatching patterns a set of 900 virtual larvae, released over four days (225 larvae d^−1^ estuary^−1^), was introduced in the surface layer near each of eight estuarine systems (Betanzos, Noia-Muros, Vigo, Minho, Cávado, Aveiro, Mondego and Tejo; Figure 1), every fortnight during nocturnal ebb tides, from February to July of 2006 and 2007. The position, age and probability of death of a total number of 86400 larvae per year (900 larvae/estuary×12 release periods×8 estuaries) were stored with a time step of two hours. To decrease computation time we only used estuaries separated by at least 40 km. Additionally, according to initial trial simulations larvae hatched from estuaries beyond Betanzos and Tejo would not recruit to the Ria de Aveiro, and estuaries beyond these were not included in the simulations. Because estuarine inlets were not conveniently represented by the model we released and recruited larvae in an area of the shelf adjacent to each estuary (approximate 12×12 km), which roughly corresponds to the area of the influence of the estuarine plume, and defined supply to the estuaries as the number of particles that crossed that respective area (Figure 1C). Tides were not used in the model because previous studies have shown that tidal currents do not affect the net horizontal advection of larvae that undergo DVM over the west Iberian shelf [51].

### Model experiments and validation strategy

We used a stepwise approach in order to validate the IBM model and to test its response to different behavioural, growth and mortality scenarios, progressing from the least constrained to the most restrictive scenario in terms of potential dispersal distance.

DVM has the potential to strongly affect cross-shelf distribution of larvae and other zooplankton in coastal upwelling systems [39], [52], by positioning these organisms in a less advective environment close to the bottom during the day. Therefore, we first examined the influence of DVM on the cross-shore distribution of larvae and retention of larvae on the shelf, by running two Base experiments that did not include mortality or any type of estuary-reinvasion behaviour, one without DVM and the other with DVM. These Base experiments used the growth rates directly estimated from published data. We compared predictions of the model against data obtained during the Heincke 09 cruise [50], which sampled 6 transects extending from the inner shelf to *ca.* 175 km offshore between 40.0 N and 41.5°N, in April of 1991, using oblique hauls by a multi-net that was towed from the surface to within 5 m of the bottom or to a 200 m depth level. As a measure of the predicted distribution of larvae during the whole planktonic development period, we calculated distributions of larval trajectory densities as the accumulated number of larvae that crossed each vertical array of cells in the model in a section delimited by the 40.0 N and 41.5°N parallels. The trajectory densities were then correlated with the cross-shelf abundance of all pooled *Carcinus maenas* larval stages collected during the Heincke 09 cruise, normalized by area of sea surface. The Base model with DVM predicted abundance maxima on the middle shelf, matching the observed distribution of larval density [50]. In contrast, the Base model without DVM predicted that very few larvae would remain in shelf waters after the expected PLD, which departed strongly from the observed distribution. We therefore developed the subsequent steps building on the Base model with DVM.

In the Base experiments we did not recruit the megalopae to the estuaries, but kept them in the model and recorded them every time they crossed the area adjacent to the Ria de Aveiro. Under this option we were interested in the potential trajectories of the larvae in the shelf, without imposing any behavioural constraint regarding proximity of the estuaries. Animations produced from the model indicated that the larvae that recruited to the Ria de Aveiro and other estuaries never left the inner shelf and had trajectories parallel to the shore, increasing the probability of recruitment of competent megalopae to estuaries. This led us to the Invasion experiments, where the megalopae were made to recruit to each estuary once they crossed the respective adjacent shelf area (i. e. the megalopae entered the estuaries and were not counted afterwards). Dispersal distance is partly correlated with PLD [53]. Because of the uncertainty of larval development times estimated from laboratory rearings, in a first set of Invasion experiments we tested normal, fast and slow growth rates, where the normal growth rate was that estimated from the literature data, and the fast and slow growth rates were obtained by arbitrarily accelerating or decelerating growth by 1 d per larval stage (*ca.* 20% of the total estimated PLD). Comparison between the predicted and observed time series of supply using cross-correlation indicated that changes in growth rate did not consistently increase or decrease fit in the two years. A further refinement of growth rate was therefore not attempted.

Mortality was introduced in a second set of Invasion experiments, separately for each of the growth rates tested previously. Because mortality was modelled as a random process, the output of the model would always change with each simulation, independently of the predicted oceanography. In order to incorporate this variability we ran the offline IBM model three times for each growth rate. Cross-correlation analysis of pairs of time series produced by each of the three runs per growth rate were consistently above 0.99 at 0 d time lags (p<0.001), and we therefore averaged the outputs of the three runs in order to compare the predictions of the Invasion experiments with mortality with the observed times series of supply.

### Statistical analysis

The adjustment of the predicted time series relative to the observations was assessed using cross-correlation analysis [54], by lagging the observed relative to the predicted series. This procedure produces negative lags when predicted maxima precede observations, and positive lags when observed maxima precede predictions. In order to reduce noise and remove strong autocorrelation at 1 d lag in both predictions and observations the series were smoothed, with a moving average of period 5 d, and differenced at a lag of −1 d. Predicted time-series from experiments with mortality were treated similarly before being cross-correlated.

## Results

### Observed time series of megalopal supply

**Figure 2:**
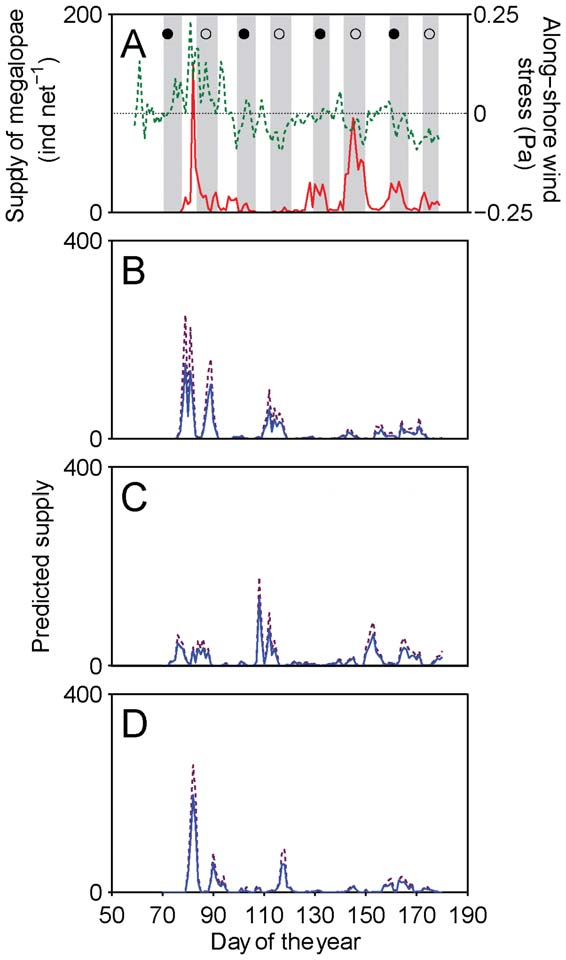
Observed and predicted time series of supply in 2006. (A) Daily numbers of observed megalopal supply (continuous line), along-shore wind stress (dashed line) and spring tides (tidal range larger than long-term average range, grey bars) in the Ria de Aveiro in 2006; and predicted time series of supply by the Invasion experiments with (B) normal, (C) fast and (D) slow growth rates with (dashed line) and without (continuous line) mortality, for the same year.

**Figure 3:**
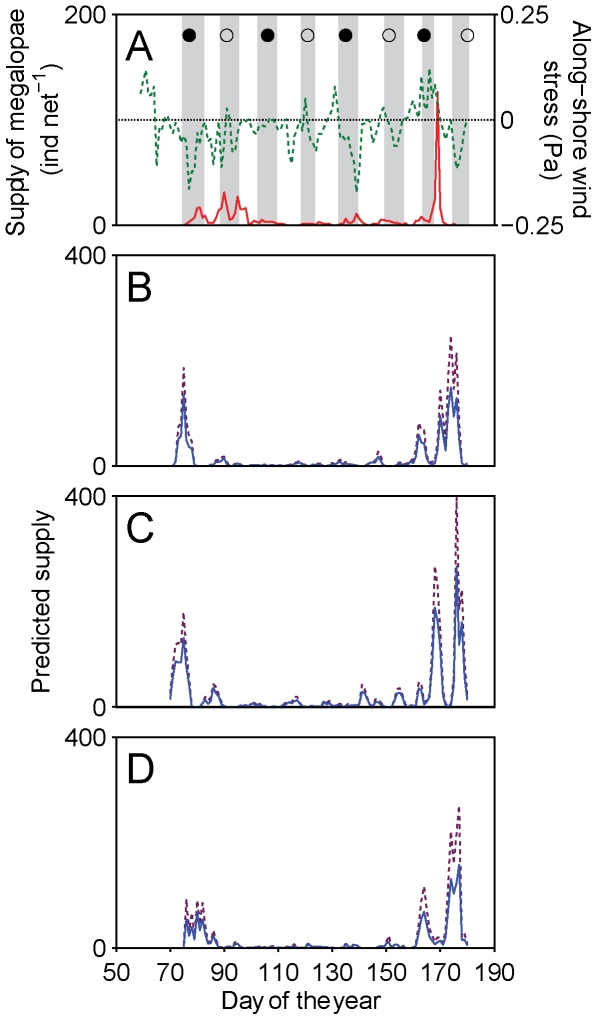
Observed and predicted time series of supply in 2007. (A) Daily numbers of observed megalopal supply (continuous line), along-shore wind stress (dashed line) and spring tides (tidal range larger than long-term average range, grey bars) in the Ria de Aveiro in 2007; and predicted time series of supply by the Invasion experiments with (B) normal, (C) fast and (D) slow growth rates with (dashed line) and without (continuous line) mortality, for the same year.

Observed supply of *Carcinus maenas* megalopae to the Ria de Aveiro in 2006 and 2007 (Figures 2A and 3A) showed periods of several weeks with zero or very low supply punctuated with supply events driven by along-shore winds, as well as several instances when supply events occurred with semilunar periodicity during spring tides in spring and summer, particularly in 2006. In both years the highest supply events followed several days of strong southerly winds and coastal convergence [40].

### Influence of DVM on cross-shelf distribution of larval trajectories in the Base experiments

**Figure 4:**
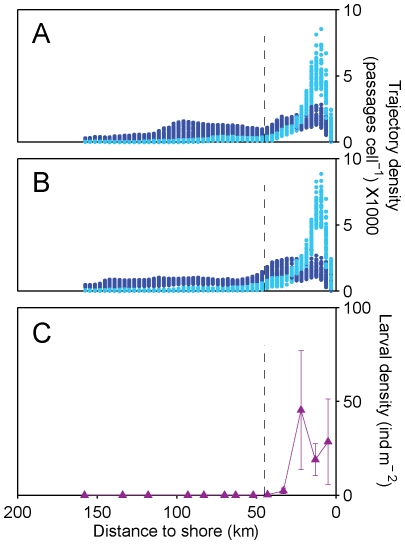
Predicted trajectory density and observed larval abundance in coastal waters. Onshore/offshore distribution of trajectory density predicted by the Base experiments with (light blue) and without (dark blue) diel vertical migration for (A) 2006 and (B) 2007; and (C) abundance of larvae (all stages combined) recorded by the Heincke 09 cruise in April of 1991, normalized by area of sea surface. Trajectory density was calculated between the parallels 40°00′ and 41°30′N, which was the area sampled by the cruise. The broken line in each panel represents the average position of the shelf break at ca. 43 km from shore.

The Base experiments without DVM resulted in very high levels of larval wastage from the shelf, compared with those with DVM, with a decrease of one order of magnitude in supply and only a few tens of particles successfully recruiting to the Ria de Aveiro. The cross-shelf distribution of trajectory densities predicted by the Base experiments with DVM, for both years, fit several features of the observed cross-shelf distribution of larval densities recorded during the Heincke 09 cruise [50], contrary to the Base experiments without DVM (Figure 4). The observations indicate maximum densities in the inner shelf, a rapid decline to very low values at *ca.* 35 km from the shore and near-zero values off the shelf break, similarly to the predictions of the Base experiments with DVM. Bonferroni-corrected Pearson correlations of log-observed average abundance of larvae with log-predicted average trajectory density were positive and significant in the case of the simulations with DVM (r = 0.82, p<0.001 and r = 0.79, p<0.01, in 2006 and 2007) and non-significant when DVM was not included (r = 0.33, p>0.25 and r = 0.05, p>0.85, in 2006 and 2007).

### Time series of supply from the Invasion experiments without mortality

An additional feature of the Base experiments was that they produced wide bimodal peaks around periods of maximum observed supply, caused by successive passages of individual competent larvae in the shelf area adjacent to the Ria. This is an unrealistic behaviour because these larvae are expected to recruit to the estuaries in natural conditions and leave the shelf. This feature was not present in the Invasion experiments with the normal growth rate, which tended to produce single narrow supply peaks within ±6 d of the high supply peaks associated with southerly winds, in March 2006 and June 2007 (Figures 2B and 3B). As expected from the fact that the model did not simulate tides or tidal behaviour, the time series of predicted supply did not reproduce the semilunar components present in the observations in 2006 after day 120. Additionally, the model produced a supply event after day 110 that was not present in the observations (Figure 2B). The long period with very low observed supply from days 95 to 165 in 2007, however, was well represented by the model (Figure 3B). In general, the Invasion models with the normal growth rate tended to predict higher supply during periods of elevated observed supply, and cross-correlation values between observations and predictions were positive and significant (0.66 and 0.63 in 2006 and 2007, p<0.05, with time lags of +5 and −3 d respectively; Table 1).

**Table 1:** Cross-correlations and time lags between observations and predictions.

Invasion model experiment	2006	2007
No mortality, fast growth rate	0.37 (−4)	0.76 (−5)
No mortality, normal growth rate	0.66 (+5)	0.63 (−3)
No mortality, slow growth rate	0.81 (+2)	0.68 (−6)
With mortality, fast growth rate	0.34 (−4)	0.73 (−5)
With mortality, normal growth rate	0.66 (+5)	0.58 (−3)
With mortality, slow growth rate	0.81 (+2)	0.65 (−6)

Cross-correlations (*r*) and time lags (d, in brackets) between observations and predicted time series, for the different Invasion model experiments and years. Reported values refer to maximum cross-correlations in each case, which were always significant at the 5% level.

Changes in growth rates produced inconsistent results (Figures 2C, 2D, 3C, 3D, Table 1). In 2006, simulations with the normal growth rate predicted supply with a delay of +5 d, whereas slow growth reduced the time gap to only +2 d and fast growth anticipated supply by −4 d. In 2007, the normal growth rate predicted supply with an anticipation of −3 d, while fast and slow growth rates also anticipated supply, with delays of −5 and −6 d. The largest cross-correlations were obtained with the slow and fast growth rates in 2006 and 2007 (0.81 and 0.76 respectively, p<0.05; Figures 2D and 3C). An analysis of PLD of the different larval cohorts to the fluctuating temperature regime that they experienced in the model (not shown) did now show any clear pattern, and this inconsistency could not be explained.

### Time series of supply from the Invasion experiments with mortality

Because changes in growth rate did not consistently result in anticipations or delays of predicted supply, nor in higher cross-correlations, it was not possible to elect a best model. Therefore, we examined the effect of mortality from sub-optimal conditions of temperature and salinity for all growth rates in a second set of Invasion experiments. In all experiments, the three runs made to examine the effect of different realizations of mortality produced pair-wise cross-correlations >0.99 at lime lags of 0 d (p<0.001), indicating that mortality from temperature and salinity does not have the potential to substantially affect the temporal patterns of supply in the NW Portuguese coast. However, mortality did lower the number of larvae that recruited successfully relative to the corresponding Invasion experiments without mortality by about 30% (Figures 2 and 3, panels B to D).

Time lags between observations and predictions were the same as those reported in the Invasion experiments without mortality (Table 1), again indicating inconsistent effects of growth rate on supply. The largest cross-correlations were also obtained with the slow and fast growth rates in 2006 and 2007 (0.81 and 0.73 respectively, p<0.05; Figures 2D and 3C).

### Dispersal pathways of recruiting and non-recruiting larvae and mortality rates

In all experiments with DVM (Base and Invasion) there was a remarkable difference in the pathways of the larvae that recruited to the Ria de Aveiro (or to the other estuaries), which were very seldom advected beyond the middle shelf (100 m isobath) and had trajectories clearly dominated by an along-shore component, relative to the larvae that never recruited, which were consistently advected beyond the middle shelf. Examples of the time course of the advection processes and supply can be seen in the animations produced with daily distributions of larvae predicted by the model in the Invasion experiments with normal growth rate and no mortality for both years (see Supporting Information Videos S1 and S2). The animations also show that the large supply peaks associated with southerly winds occur during periods of onshore convergence and northward flow.

Estimates of survival to successful recruitment ranged from 10 to 17% of the total number of larvae hatched from all estuaries, with an average of 13%. Pooling years, recruitment rate increased with increasing growth rate (10, 13 and 16% for slow, normal and fast growth rates, respectively). Pooling growth rates, recruitment rate was 12% in 2006 and 14% in 2007. Mortality due to physiological stress, estimated from the percentage of the total number of larvae that died from this cause in the Invasion models that include mortality, ranged from 34% to 40%, averaging 37%. Pooling years, there was a consistent decrease in this component of mortality with growth rate (34, 37 and 40%, for slow, normal and fast growth rates, respectively). Pooling growth rates, mortality was identical in the two years (37%). Larval wastage, estimated from the complement of physiologic mortality plus recruitment, changed little with growth rate and year, averaging 50% (50% for all growth rates when pooling years; and 50% and 49% in 2006 and 2007, respectively, when pooling growth rates).

### Dispersal distance

**Figure 5:**
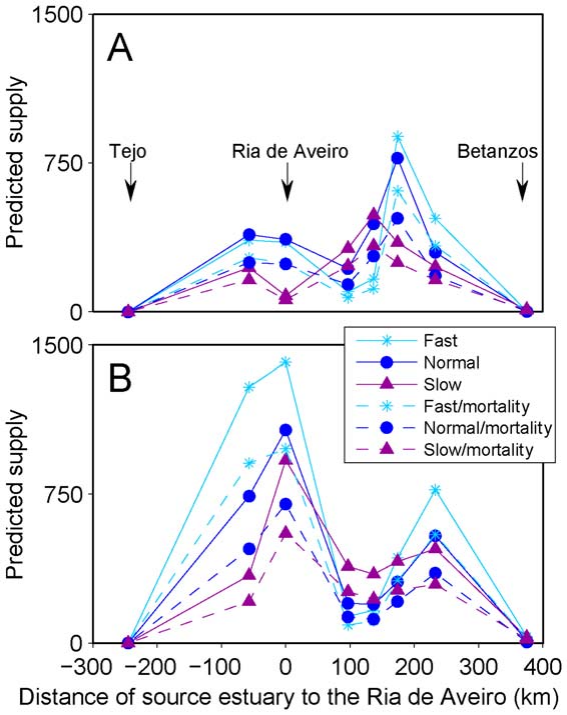
Predicted supply of each source estuary to Ria de Aveiro. Average daily contribution of each estuary to the Ria de Aveiro as a function of distance to source estuary predicted by the Invasion models for (A) 2006 and (B) 2007.

The number of larvae predicted to recruit to the Ria de Aveiro by the Invasion models, as well as the contribution of each source estuary to the Ria, changed with year, growth rate and mortality (Figure 5). In 2006 northern estuaries contributed more to the Ria than southern estuaries, but this pattern reversed in 2007. Self-recruitment to the Ria was also about 3 times higher in 2007. All Invasion experiments predicted that estuaries from the Mondego to Noia supplied larvae to the Ria de Aveiro. The Tejo estuary never contributed to supply to the Ria. Invasion experiments predicted a weak supply from Betanzos, except those with mortality in 2006 that predicted no supply from this estuary. Maximum realized dispersal distance (i.e., dispersal distance of successfully recruiting larvae, negative from south, positive from north) therefore depended on the direction of dispersal and on the inclusion of mortality in the models. Maximum realized dispersal of larvae supplied from south was constant at −57 km (all larvae originated from the Mondego estuary) whereas maximum realized dispersal of larvae supplied from north was 377 km or 232 km depending on whether there was supply from Betanzos or not.

**Figure 6:**
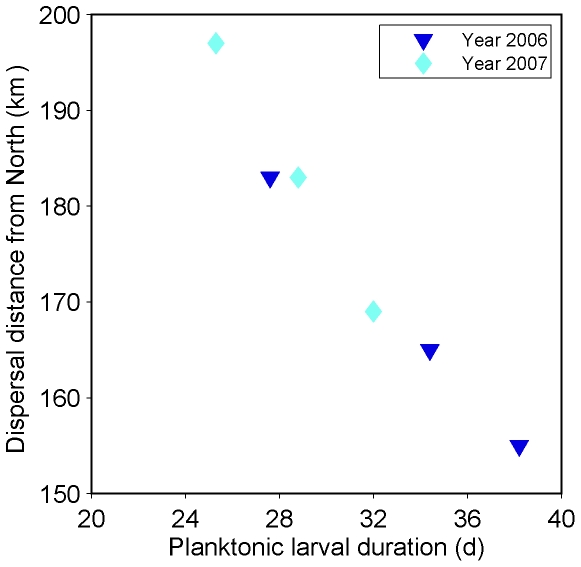
Effect of PLD on larval distance. Average dispersal distance of successfully recruiting larvae supplied to the Ria de Aveiro from northern estuaries as a function of planktonic larval duration predicted by the Invasion models.

Despite these trends, the estimates of the average realized dispersal of successfully recruiting larvae did not change much (Table 2). Larvae supplied from southern estuaries always dispersed 57 km, as they all originated from the Mondego estuary. Average dispersal distances of larvae supplied from northern estuaries ranged from 155 to 198 km, with an average of 175 km. Mortality had a very small effect on average realized dispersal decreasing it only by 1 km in the experiment with slow growth rate in 2007. On the contrary, growth rate had a consistent effect on dispersal distance when larvae were supplied from multiple estuaries (those located to the north of the Ria de Aveiro), which increased from slow to fast growth rates. This is evident by the strong negative correlation between dispersal distance by larvae supplied from the north and age at recruitment (both years, all growth rates; Figure 6), which was 0.98 (p<0.001, n = 6).

**Table 2:** Estimates of the average realized dispersal of successfully recruiting larvae.

Invasion model experiment	2006		2007	
	From South	From North	From South	From North
No mortality, fast growth rate	−57	183	−57	197
No mortality, normal growth rate	−57	165	−57	183
No mortality, slow growth rate	−57	155	−57	169
**Average**	−57	168	−57	183
With mortality, fast growth rate	−57	183	−57	198
With mortality, normal growth rate	−57	165	−57	183
With mortality, slow growth rate	−57	155	−57	168
**Average**	−57	167	−57	183

Average dispersal distance (km) of successfully recruiting larvae (realized dispersal) for the different Invasion model experiments and years.

## Discussion

The Base experiment without DVM predicted that the larvae would be advected, on average, to distances as far as 200 km off the coast. These predictions depart strongly from observed field distributions of *Carcinus maenas* larval stages, which show the highest concentrations within 22 km from shore and no larvae present beyond the shelf break [38], [50]. Importantly, field observations indicate that *Carcinus maenas* larvae do display strong nocturnal vertical migration behaviour [38] and a previous modelling attempt indicated that, for a vertically migrating larvae, upwelling could be beneficial for retention given the time that larvae would spend in the compensating bottom undercurrent [39]. This suggests that DVM may limit dispersal in coastal environments that experience upwelling, a conclusion supported by the present study as all modelling experiments with DVM showed that successfully recruiting larvae never left the inner shelf. Overall, the dispersal patterns obtained with DVM are consistent with recent findings from other upwelling systems indicating that larval development of many coastal invertebrates takes place in the inner shelf environment [55].

The prediction that most of the non-periodic changes in larval supply results from along-shore advection is a major suggestion of the model. Along-shore circulation has been associated with changes in supply and recruitment of coastal invertebrates by interacting with coastal topography and redistributing the larvae regionally. For instance, crab and sea urchin larvae in the upwelling system of northern California larvae trapped in the lee of Point Reyes and in the Gulf of the Farallones are released following relaxation of upwelling winds, resulting in transport of larvae and increased settlement to northward locations [56]. We suggest that the mechanism operating on the northwest coast of Iberia has a different nature. In the stretch of coast where larvae recruiting to the Ria de Aveiro develop, which extends from the Estremadura promontory to Cape Finisterre, there are no major capes nor gulfs that could act as larval traps. Instead, the location of larval pools along the coast and the delivery of competent larvae to settlement habitats depend on local larval production, growth and alternating along-shore advection history. The positive or negative time lags obtained in the various Invasion experiments likely arise from the topographically un-trapped pools and the current uncertainty in larval growth rates and the location, timing and magnitude of larval production events. This pattern of advection and the inner-shelf retention of a significant fraction of the larval pool also suggest a conceptually new mechanism for estuarine reinvasion of crab megalopae. Instead of being exclusively dependent on mechanisms for cross-shore advection, competent megalopae have an increased probability of being mixed into the estuarine plumes during along-shelf dispersal. Megalopae of estuarine crabs have been shown to react to estuarine water by arresting swimming and dropping to the bottom under daylight conditions [57]. This reaction results from the inhibitory effect of chemicals present in estuarine water produced by the decomposition of organic matter. The successive passage of megalopae close to estuarine inlets and the effect of chemical cues could result in the accumulation of competent larvae in the bottom close to estuaries and provide a trapping mechanism that would enhance supply into estuaries during night flood tides [58], [59], [60], [61].

In the Invasion experiments, mortality caused by physiological stress from temperature and salinity averaged 37%, while wastage was around 50%. Mortality did not affect predictions of average realized larval dispersal because there are no strong temperature gradients in the area to which dispersal is constrained, and the observed and predicted salinity values are widely tolerated by *Carcinus maenas* larvae [34]. Mortality did lower maximum realized dispersal distance by reducing to zero the contribution of the distant populations, a consequence that is acknowledged by theoretical [62] and modelling [13], [63] studies. The effects of mortality on larval dispersal and connectivity derived from modelling studies, however, should be approached with care, because the number of larvae that the models are able to simulate is many orders of magnitude lower than the number of propagules actually produced by most marine populations, and the thinning effect of mortality may not be felt in distant sources because dispersal is actually constrained by the physics of the ocean, which limits dispersal and sets a maximum distance at which the farthest sources can be located.

An unexpected prediction of the modelling experiments was that the dispersal distance of successful recruiting larvae supplied from northern estuaries decreased with increasing PLD. It is generally considered that realized dispersal distance increases as PLD increases, and comparative empirical evidence available from a variety of species with contrasting life history traits supports this view [53]. The counter-intuitive result obtained in the present study derives from the increase in physiologic mortality rate with longer PLD, a well-known effect [12] that was captured by the model, which decreased the chance of successful recruitment from distant sources. The suggestion that, in the environment where marine larvae disperse, travelling fast in short time intervals may be the best way to travel far may be pertinent for other species and oceanographic regimes and may explain part of the variability in intra- and inter-specific realized distance.

The estimates of average realized dispersal were −57 and 175 km (for larvae supplied from southern and northern estuaries, respectively). The net result of dispersal along the western Iberian shelf would therefore be southwards, which is consistent with the dominance of upwelling events during spring and summer [64]. Besides successfully predicting the observed cross-shelf distribution of larvae, the predictions of the model are remarkably consistent with estimates of average larval dispersal distances for *Carcinus maenas* based on the range increase of the species in invaded ecosystems (63 and 173 km) [53], with observations of genetic homogeneity among populations from the Gulf of Cadiz, South Spain, to Wales, UK, a range of approximately 3000 km [8], and with the lack of sweepstakes reproduction in the species [65]. Since the model predicts maximum dispersal distances of 377 km from the north and 57 km from the south, homogenization of allele frequencies along this 3000 km range must take several generations. The model also predicts that each larval event is a mixture of larvae from different estuaries and therefore it lends support to the observations of no genetic differences among supply events and between larvae and the adult population found previously [65].

Modelling approaches that simultaneously include key physical dynamics and biological traits provide a way forward to investigate dispersal and connectivity of marine populations. In order to increase their broad applicability, biophysical models need further development in order to resolve several key issues such as two-way coupling across different scales when using nested models, near-shore and other boundary currents, non-hydrostatic flows, forcing factors at event time scales and sub-grid scale parameterizations, as well as a better understanding of larval behaviour, mortality factors and other biological traits [66]. A limitation of the present model is the difficulty in resolving processes in the sticky near-shore zone, which increasingly seems to be relevant for littoral species [67], that is caused by constraints imposed on grid size and by the inability of hydrostatic models such as ROMS to deal with waves. In the present case however one can argue that this does not seriously affect the predictions of the model, since *Carcinus maenas* larvae are ejected from estuaries with the estuarine plume and will likely escape the very near-shore. Other limitations with a greater potential to affect the fit of the model are the absence of tidal behaviour in the individual based model, and of seasonal or spatial variation in larval hatching. Therefore, the model cannot reproduce semi-lunar or seasonal patterns of larval reinvasion and supply, nor incorporate the interaction between spatially and temporally changing reproductive outputs [68].

In spite of these limitations, the biophysical model used in the present study appears to provide realistic estimates of the spatial and temporal scales of the dispersal driven by along-shore winds, which is the main factor regulating variability of oceanic currents in upwelling continental shelves. The use of this model also enabled us to link descriptions of the dispersal and supply processes, which so far have been addressed independently given the challenges imposed by the disparate spatial and temporal scales involved [15], [66]. Finally, we wish to note that this study indicates that, provided the relevant information is available, spatial and temporal patterns of larval dispersal and supply can be described by mechanistic bio-physical models, challenging the paradigm that dispersal and recruitment in marine populations is essentially a stochastic phenomenon [69].

## Supporting Information

Video S1 Time evolution of larval trajectories for the complete larval series (first zoea to megalopa) for the Invasion experiment with normal growth rate without mortality in 2006. Left panel: trajectories of the larvae that never recruited to the Ria de Aveiro, right panel: trajectories of the larvae that recruited to the Ria de Aveiro. Arrows close to estuaries indicate periodic hatching of larvae and moving arrows represent direction and intensity of the wind. Larvae are colour-coded according to natal estuary and become grey at moult to megalopae. Red, blue and green flashing circles indicate recruitment events in the observations and the model, in the observations only, and in the model only, respectively. Recruitment events are defined as when daily observed and predicted supply of megalopae exceeded average levels for at least 3 consecutive days.
(AVI)

Video S2 Time evolution of larval trajectories for the complete larval series (first zoea to megalopa) for the Invasion experiment with normal growth rate without mortality in 2007. Left panel: trajectories of the larvae that never recruited to the Ria de Aveiro, right panel: trajectories of the larvae that recruited to the Ria de Aveiro. Arrows close to estuaries indicate periodic hatching of larvae and moving arrows represent direction and intensity of the wind. Larvae are colour-coded according to natal estuary and become grey at moult to megalopae. Red, blue and green flashing circles indicate recruitment events in the observations and the model, in the observations only, and in the model only, respectively. Recruitment events are defined as when daily observed and predicted supply of megalopae exceeded average levels for at least 3 consecutive days.
(AVI)
